# Supplementary material for: Geobacter sulfurreducens’ Unique Metabolism Results in Cells with a High Iron and Lipid Content
Source: Microbiol Spectr. 2022 Oct 27;10(6):e02593-22. doi: 10.1128/spectrum.02593-22 (PMC9769739; doi:10.1128/spectrum.02593-22)
Supplement: Supplemental File 1 — Supplemental material. Download spectrum.02593-22-s0001.pdf, PDF file, 0.3 MB [file spectrum.02593-22-s0001.pdf]

Supporting Information

Table S1. Chemicals in Ringer's solution per L of medium used. One fourth strength of Ringer's solution was used for washing cells.

Chemicals	Amount (g)	Concentration (mM)	¼ Strength Ringer's solution Conc. (mM)
NaCl	6.5	111	27.8
KCl	0.42	5.6	1.41
CaCl2	0.25	2.3	0.56
NaHCO3	0.2	2.4	0.6

Table S2. Media composition in different conditions tested.

Species	*Geobacter sulfurreducens* PCA				*Escherichia coli* K-12			
Growth condition	Electrode		Fumarate		G medium		M9 medium	
Unit	g in 1 L	mM	g in 1 L	mM	g in 1 L	mM	g in 1 L	mM
$NaCH_3COO$ $3H_2O$	6.8	50	6.8	50	-	-	-	-
NaFumarate	-	-	16	100	-	-	-	-
Glucose	-	-	-	-	8	44	8	44
$NaHCO_3$	2.5	30	2.5	30	2.5	30	-	-
NaH_2PO_4	0.6	4	0.6	4	0.6	4	25.6	180
KH_2PO_4	-	-	-	-	-	-	6	44
KCl	0.1	1	0.1	1	0.1	1	-	-
NaCl	-	-	-	-	-	-	1	17
NH_4Cl	1.5	20	1.5	20	1.5	20	2	37
$MgSO_4$ $7H_2O$	-	-	-	-	-	-	0.049	0.200
$CaCl_2$	-	-	-	-	-	-	0.001	0.010
Thiamin	-	-	-	-	-	-	0.001	0.024
$FeCl_2$ $4H_2O$	0.004	0.020	0.004	0.020	0.004	0.020	-	-
Na_2S $9H_2O$	0.013	0.054	0.013	0.054	0.013	0.054	-	-
vitamin mix	10 mL	10 mL	10 mL	10 mL	10 mL	10 mL	-	-
trace mineral*	10 mL	10 mL	10 mL	10 mL	10 mL	10 mL	-	-

* Trace mineral stock solution in 1 L

Trace mineral	Al	B	Ca	Cl	Co	Cu	Fe*	K*
mg in 1 L	0.006	0.014	0.273	7.595	0.248	0.025	0.201	0.008
Trace mineral	Mg	Mn	Mo	Na*	Ni	S*	W	Zn
mg in 1 L	2.958	1.625	0.107	3.989	0.059	5.656	0.014	0.624

Table S3. Metal content in dry cell mass of *Geobacter sulfurreducens* PCA and *Escherichia coli* K-12 (unit: milligram of each metal per gram of dried bacterial cell, mg/gdw).

	G. sulfurreducens		***E. coli***	
	Anode	Fumarate	M9 medium	*Geobacter* medium
Ag	0.098 (± 0.073)	0.106 (± 0.034)	ND	ND
Ba	0.028 (± 0.002)	0.025 (± 0.003)	0.021 (± 0.005)	0.027 (± 0.003)
Co	0.028 (± 0.005)	0.021 (± 0.029)	ND	0.003 (± 0.002)
Cr	0.081 (± 0.016)	0.086 (± 0.014)	0.006 (± 0.003)	0.003 (± 0.000)
Cu	0.224 (± 0.086)	0.471 (± 0.107)	0.017 (± 0.005)	0.048 (± 0.010)
Fe	1.970 (± 0.226)	1.96 (± 0.229)	0.134 (± 0.053)	0.428(± 0.089)
Li	0.047 (± 0.030)	0.017 (± 0.001)	0.040 (± 0.003)	0.047 (± 0.006)
Mg	0.67 (± 0.27)	0.54 (± 0.11)	2.07 (± 0.75)	1.79 (± 0.38)
Mn	0.583 (± 0.076)	0.324 (± 0.067)	0.023 (± 0.021)	0.570 (± 0.122)
Ni	0.069 (± 0.012)	0.076 (± 0.012)	0.002 (± 0.003)	ND
Pb	0.036 (± 0.015)	0.023 (± 0.003)	0.018 (± 0.011)	0.016 (± 0.010)
Se	0.104 (± 0.058)	0.177 (± 0.112)	0.012 (± 0.005)	0.101 (± 0.016)
Sr	0.008 (± 0.002)	0.017 (± 0.006)	0.005 (± 0.003)	0.003 (± 0.001)
V	0.002 (± 0.001)	0.003 (± 0.002)	0.035 (± 0.006)	0.045 (± 0.005)
Zn	3.5 (± 1.9)	10.0 (± 2.4)	0.135 (± 0.034)	0.176 (± 0.024)

Table S4. Estimated growth of *G. sulfurreducens* per L of medium at an anode based on the available mineral concentrations in Geobacter medium.

Metal	*Geobacter* Medium [mg element/L]	Average *G. sulfurreducens* content (mg element/g cell)	Estimated growth (g cells/L)

Mg	2.958	0.67	4.42
Mn	1.625	0.583	2.79
Fe	0.201	1.97	0.10
Co	0.248	0.028	8.80
Zn	0.624	3.5	0.18
Cu	0.025	0.224	0.11
Ni	0.059	0.069	0.86

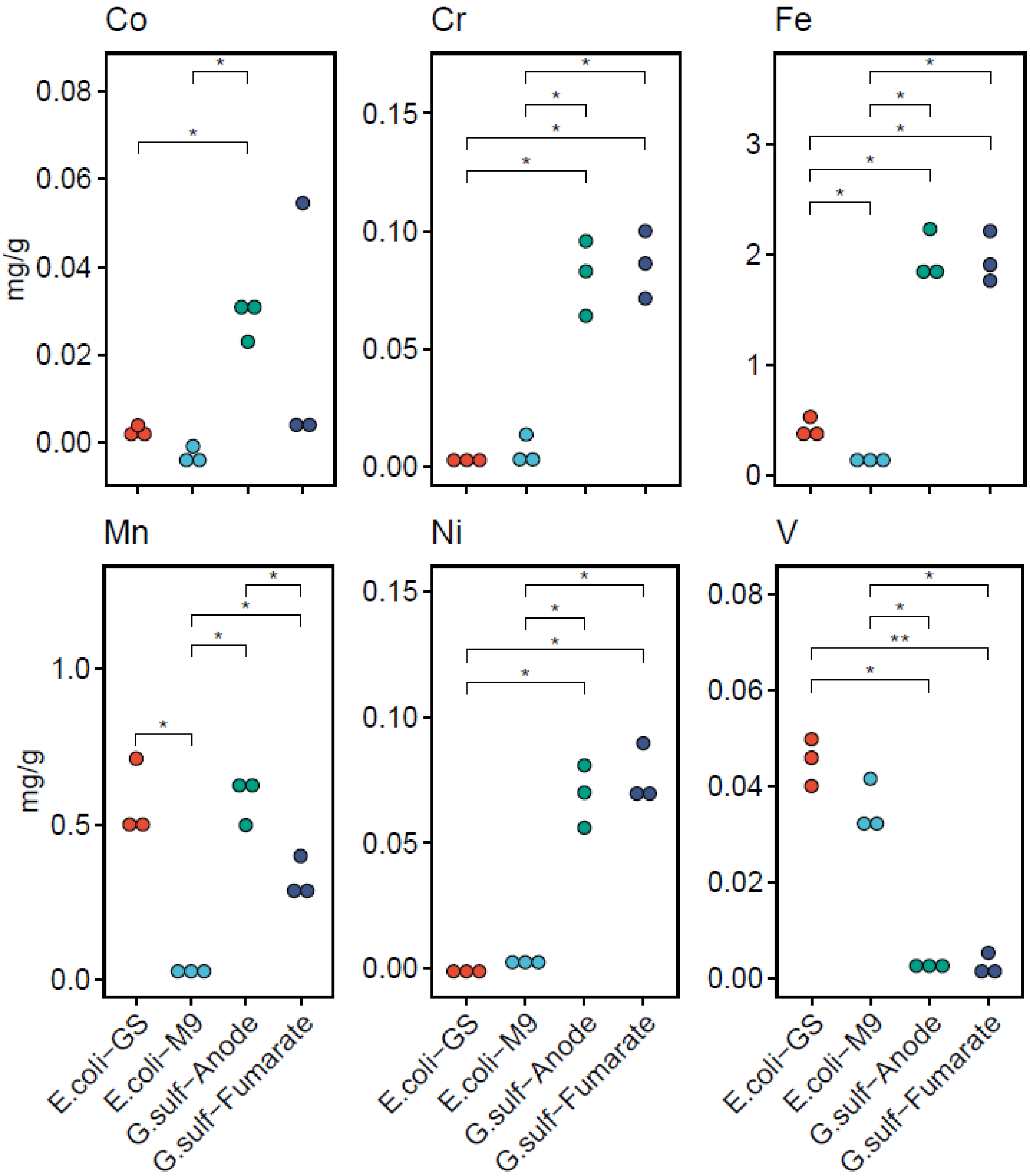

Figure S1. Statistically significant differences in metal concentrations between *E. coli*-GS (Geobacter medium), *E. coli*-M9 (M9 medium), *G. sulf*-Anode (biofilm grown on an electrode), and *G. sulf*-Fumarate (planktonic cells using fumarate as the electron acceptor). *(p<0.05), **(p<0.001), pairwise t-test with multiple comparison correction performed with the Benjamini-

Hochberg method. We chose to omit alkali metals from this figure, but Lithium did have significant differences as well (Table S3).

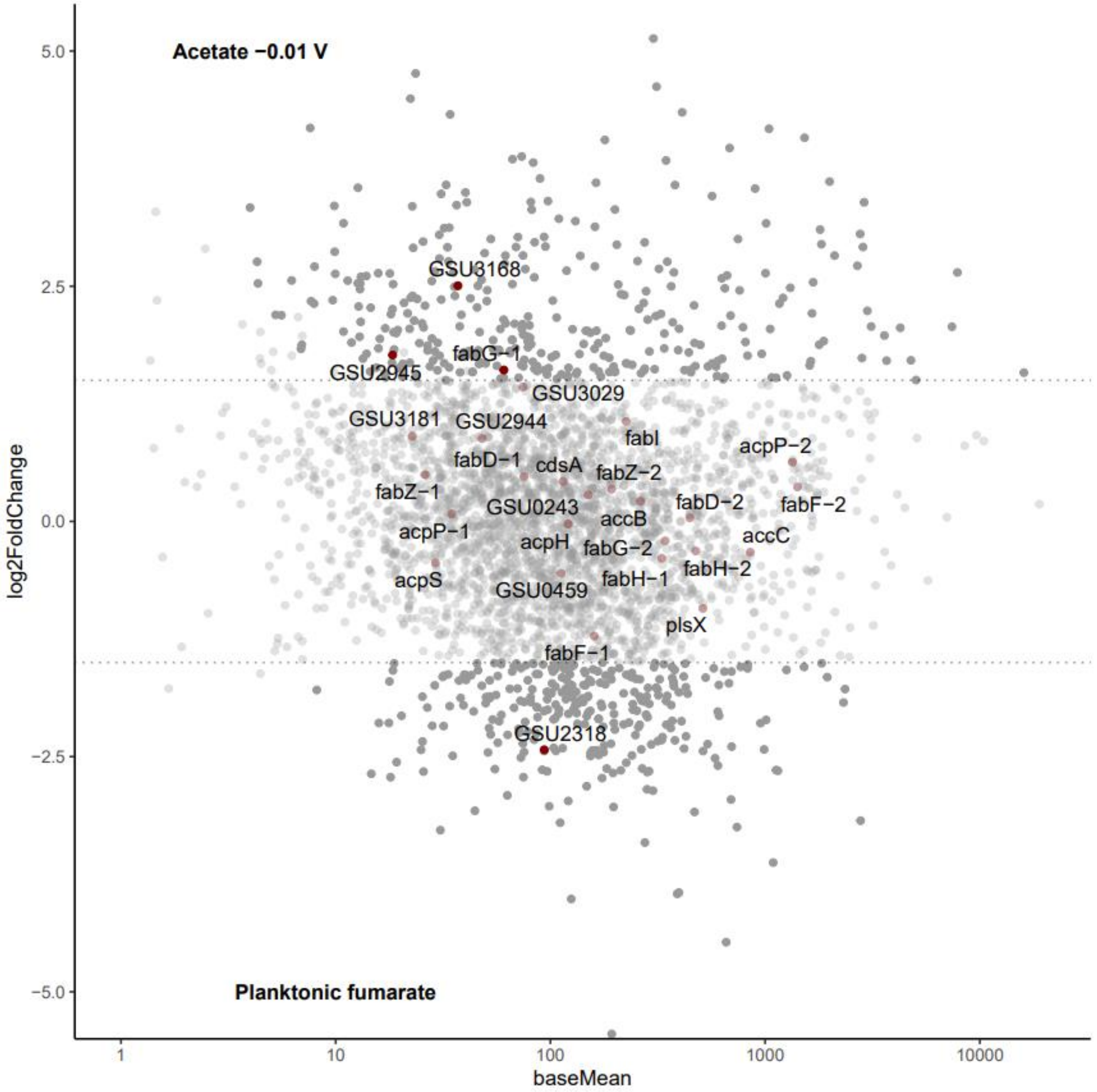

Figure S2: MA plot with lipid synthesis pathway genes annotated. Dotted lines indicate $\log_2$ fold change of 1.5, and solid dots indicate an adjusted p value under 0.05.
